# A Prospective Examination of Mental Health Trajectories of Disaster-Exposed Young Adults in the COVID-19 Pandemic

**DOI:** 10.3390/bs14090787

**Published:** 2024-09-07

**Authors:** Melissa Janson, Erika D. Felix, Natalia Jaramillo, Jill D. Sharkey, Miya Barnett

**Affiliations:** 1 Department of Psychiatry and Behavioral Sciences, University of Washington, Seattle, WA 98195, USA; 2 Department of Counseling, Clinical, and School Psychology, University of California, Santa Barbara, Santa Barbara, CA 93106, USA; 3 Department of Psychiatry and Biobehavioral Sciences, University of California, Los Angeles, Los Angeles, CA 90095, USA

**Keywords:** young adult mental health symptoms, mental health trajectories, collectively experienced traumas, natural disaster, COVID-19 pandemic, life stressors

## Abstract

This longitudinal study examines young adult mental health (MH) trajectories after exposure to natural disasters (i.e., hurricanes, wildfires, mudslides) across four waves, two pre- and two during the COVID-19 pandemic. Participants (*n* = 205) answered questions about anxiety, depression, and post-traumatic stress symptoms (PTSSs) across Waves (Ws) s 1–4 and pre-pandemic factors (prior trauma history, disaster exposure, life stressors since disaster) at Wave (W) 1. Hierarchical linear modeling was conducted to examine MH trajectories and associations with pre-pandemic factors. Only the PTSS trajectory significantly differed across all Ws, with the largest increase between Ws 2 and 3 (pre- and during-pandemic time points). Prior trauma history and life stressors since the disaster were significantly associated with all MH trajectory intercepts but not growth rates.

## 1. Introduction

Disasters or collectively experienced traumas (e.g., natural, human-caused) can cause widespread disruption, damage, and loss. Life-threatening or stressful experiences that occur during or after exposure to disasters (e.g., hurricanes, wildfires, mudslides), can have long-term impacts on physical and mental health (MH) [1,2]. Exposure to multiple disasters can have a cumulative impact on psychological functioning and is associated with increases in MH symptom severity and distress during subsequent disasters [3,4,5].

Young adults ages 18 to 30 years old may be more vulnerable to the psychological impacts of disaster exposure than older adults and reported more severe MH symptoms (e.g., anxiety, depression, and post-traumatic stress) after natural disaster exposure [6,7] and during the COVID-19 pandemic [8,9,10,11]. Despite having greater psychological impacts post-disaster, young adults have been overlooked in disaster MH research conducted pre-pandemic. Few studies have focused specifically on young adults’ psychological and other health-related outcomes post-disaster [12,13,14,15,16]. Often, in disaster MH studies, young adults are grouped together with adults of all ages; however, disasters may pose unique challenges to young adult’s MH and psychosocial development [17].

Additional research focused specifically on young adults’ experiences in disaster contexts is needed and can inform disaster prevention/intervention work and aid recovery processes. Specifically, examining risk and resilience factors associated with long-term patterns of MH among disaster-exposed young adults can enhance the prediction of outcomes [18]. Also, having prospective, pre-disaster data can increase understanding of post-disaster adaptation and recovery processes [2]. The current prospective, longitudinal study aimed to examine the MH trajectories of young adults exposed to natural disasters that occurred in 2017 and 2018; data was collected across four time points (two before, and two after the start of the COVID-19 pandemic in 2020).

### 1.1. Conceptual Foundations: Transactional-Ecological Theory and a Disaster Mental Health Model

The transactional-ecological theory of development [19] and empirical evidence supporting a conceptual model predicting MH symptoms post-disaster [20] inform the current study’s conceptualization. Individual and environmental characteristics reciprocally influence development, functioning, and symptomatology [21]; however, some exert a greater influence on individual change and development than others. Several important factors identified as influencing post-disaster MH include disaster exposure (e.g., frightening or life-threatening experiences during a disaster, experiences of loss or disruption in the aftermath); an individual’s demographics or pre-existing characteristics (e.g., race/ethnicity, age, gender identity; pre-disaster psychological functioning); and aspects of the post-disaster recovery environment (e.g., coping abilities, life stressors, social support) [20,22].

Disaster exposure and an individual’s demographic/pre-existing characteristics simultaneously influence a person’s efforts to process and cope with life stressors that have occurred since the disaster. Greater levels of disaster exposure are associated with post-disaster MH [22,23,24]. Adults who are younger in age, female, of low socio-economic status (SES), and/or identify as being a member of an ethnic/racial minority group are at risk of experiencing more severe MH symptoms than those who are older, male, of high-SES, and/or White [23,25,26]. Low-SES and ethnic/racial minority groups often live in geographical locations that are vulnerable to disasters and face significant health disparities and systemic challenges that affect post-disaster recovery processes [27,28,29]. Prior trauma history or lifetime exposure to potentially traumatic events and pre-disaster MH and/or prior history of mental illness are also associated with post-disaster MH symptom severity [2,7,20,23,30]. Characteristics of the post-disaster recovery environment include life stressors since the disaster, social support, and coping. Life stressors are considered short-term events that occur in the aftermath of a disaster (e.g., fighting with a loved one, losing a job, being displaced from housing) that may magnify stress reactions [7,20,23]. Such stressors partially explain associations between disaster exposure and MH [2] and impact coping abilities [20,24]. Coping can include a variety of helpful or maladaptive strategies, which may be associated with PTSS or other MH symptoms.

### 1.2. Broad Impacts of the COVID-19 Pandemic

In March 2020, the World Health Organization (WHO) declared the transmission of the SARS-CoV-2 (COVID-19) virus a pandemic. Around the world, everyday life was disrupted across family, educational, occupational, medical, and societal systems. Some stressors specific to the COVID-19 pandemic included stay-at-home public health orders or quarantines, work and school closures, isolation, loss of financial security or growing financial losses or strain, conflicting messaging from governmental or health organizations, inadequate responses and resources for medical workers, conflict among families, loss of loved ones due to COVID-19, inability to receive medical care, and general uncertainty of the future [31,32]. Individual demographic/pre-existing factors associated with psychological distress during the pandemic were consistent with the pre-COVID-19 disaster MH literature and included female gender, younger age (under 40), college attenders, low SES, ethnic/racial minority identity, prior MH problems, and/or past exposure to trauma or stress [33,34,35,36,37,38,39].

### 1.3. Young Adult Development and Pre- and During-Pandemic Mental Health

Young adults ages 18 to 30 in contemporary Western societies are situated in a developmental phase called emerging or young adulthood, occurring after adolescence but before being settled fully into adult life. This period is characterized by identity development, instability, self-focus, feeling uncertain or in-between, having many possibilities ahead, and numerous life transitions [40,41,42]. Young adults may explore a variety of possibilities in personal identity, values and beliefs, relationships, careers, values, and living spaces and form, break, and reform social connections as they experiment with who they are and what they want out of life [39,41]. For some, the experience of young adulthood can be perceived as exciting yet also challenging, complex, and prolonged. As young adults attempt to establish themselves or accomplish a variety of educational, professional, familial, or personal endeavors, they may experience stress and MH problems. Compared with their older counterparts, young adults reported more severe MH symptoms and stress (e.g., relating to news, work, money, educational endeavors, finances, and health concerns) prior to the COVID-19 pandemic [43,44,45,46]. Over the past few decades, self-reported rates of depression, anxiety, anger, nonjudicial self-injury, suicidal ideation, and suicide attempts among young adult university students have significantly increased [47,48]. Colleges have also reported increases in MH service utilization, indicating a growing need for psychological support [47].

During the COVID-19 pandemic, young adults continued to experience a high prevalence of stress and MH symptoms and an increase in severity levels. A third or greater of young adults in the United States (U.S.) reported clinically significant depression, anxiety, and post-traumatic stress disorder (PTSD) symptoms [11,49,50,51], and about 60% of a diverse sample of young adult university students met the criteria for at least one MH disorder [37]. Prospective longitudinal studies observed significant increases in MH symptom severity (e.g., anxiety, depression) from pre-pandemic time points to the spring of 2020 (during-pandemic) among adolescents and young adults [50,52]. From March to July 2020, young adult depression and anxiety symptoms peaked in April and decreased from May onward, resulting in a decelerating quadratic shape [49,52]; young adults with higher initial COVID-19-related concerns (e.g., fears about safety while attending school or becoming infected with the COVID-19 virus) at the start of the pandemic showed more gradual declines in symptoms than with lower COVID-19 related concerns [52].

Many factors were associated with young adult MH symptoms and distress during the pandemic. Loneliness, COVID-19-associated worry, low distress tolerance, pre-pandemic MH symptoms and social stress (e.g., bullying victimization, perceived feelings of social exclusion) were associated with during-pandemic young adult MH symptoms [49,50,51,52,53]. Many young adults also reported experiencing uncertainty about their future endeavors (e.g., education) due to the pandemic [8]. Additionally, young adults that belonged to ethnic/racial minority groups (e.g., Hispanic/Latinx) experienced more severe MH symptoms during the COVID-19 pandemic than White young adults [36]. The COVID-19 pandemic has worsened MH disparities and MH care access and utilization among ethnic/racial minority young adults and may have long-term impacts [54,55].

### 1.4. Current Study

The current study initially focused on examining the psychosocial adjustment of young adult university students exposed to natural disasters that occurred in 2017 and 2018 but was adapted to also collect data related to experiences during the COVID-19 pandemic in 2020 and 2021. Having data prior to the pandemic allowed for the opportunity to examine associations between pre-pandemic factors and during-pandemic MH symptoms. Data was collected among young adults in the mainland U.S. and Puerto Rico across four waves, two post-natural disaster/pre-pandemic and two during-pandemic. 

The current study aims were to (1) examine MH symptom trajectory patterns over time (pre- to during-pandemic), and (2) examine associations between pre-pandemic factors and MH symptom severity at Wave (W) 1 (i.e., trajectory intercept) and changes in MH symptoms between each wave (i.e., trajectory growth rates). Pre-pandemic factors were measured at W1 and include prior trauma history, natural disaster exposure, and life stressors since natural disaster. MH symptoms were assessed at Waves (Ws) 1–4 and included anxiety, depression, and post-traumatic stress symptoms (PTSS). 

MH symptom trajectories were hypothesized to decline after natural disaster exposure, significantly increase after the initial start of the pandemic, and gradually decline again, based upon pre- and during-pandemic MH symptom patterns observed in a general adolescent/young adult sample [52]. Each pre-pandemic factor (e.g., prior trauma history, disaster exposure, and life stressors since disaster) was also hypothesized to be associated with more severe MH symptoms at W1, as well as with changes in MH symptoms between each wave. 

## 2. Materials and Methods

**Figure 1:**
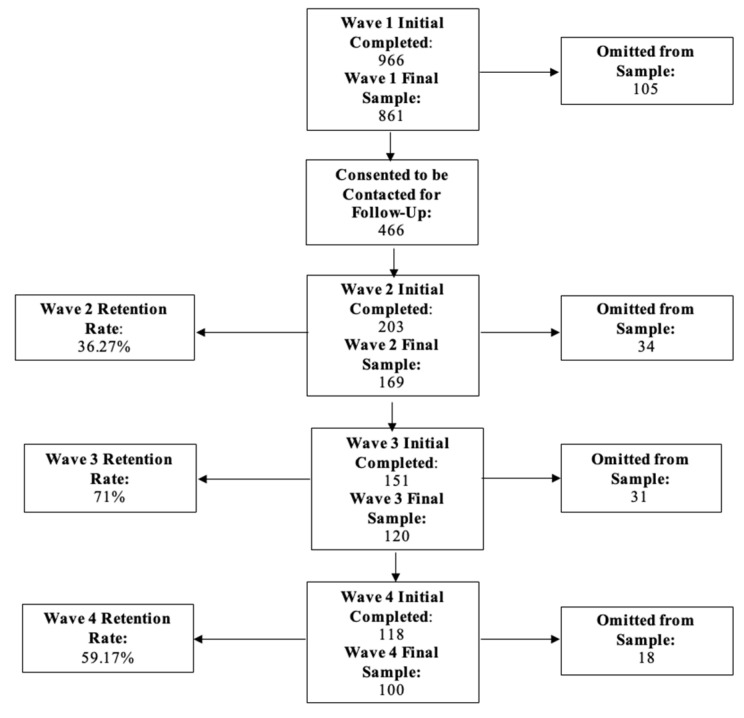
Participant flow chart across waves. Note. Participants were omitted from each wave due to incomplete data (more than 30% of the survey items were missing), survey completion under 5 min, or reported age over 30 years old. W2 retention rate was calculated by dividing 169 by 466. W3 and W4 retention rates were calculated by dividing each wave’s final sample total by the W2 final sample total, as this was considered the extended study subsample. These numbers do not reflect the same numbers of samples used to conduct study analyses and are general participation trends across the study.

Data were collected from disaster-exposed young adult university students in the mainland U.S. (California, Florida, Texas) and Puerto Rico through online Qualtrics questionnaires administered across four waves. The Wave (W) 1 survey was initially disseminated in English and then Spanish and asked about participants’ experiences and MH after exposure to natural disasters (e.g., wildfires, hurricanes) that occurred in 2017 and 2018. The W1 English survey was distributed in the mainland U.S. in the winter of 2018. After the translation of the W1 English survey to Spanish, the W1 Spanish survey was distributed in Puerto Rico in the summer of 2018. W1 survey participants (*n* = 861) were asked if they would like to be later contacted and invited to participate in subsequent waves (2–4) as part of an extended longitudinal study. A subset of W1 participants (*n* = 466) agreed to be contacted and provided personal contact information. Longitudinal data collection occurred during the following times: W2 in winter 2019, W3 in summer 2020 (during the COVID-19 pandemic), and W4 in spring 2021. The participant sample experienced attrition over time (see Figure 1 for details on the number of participants per wave). For the completion of the W1 survey, participants chose to receive research participation credit through their universities or to enter a raffle to win one of many (20) e-gift cards (USD 25.00). For the completion of the Ws 2–4 surveys, each participant received an e-gift card of USD 5.00 per wave. The subsample of 205 participants who completed W1 and one or more of the subsequent three waves was used to evaluate the current study’s aims.

### 2.1. Participants

Disaster-exposed young adults (*n* = 205) in mainland U.S. and Puerto Rico completed surveys in English and Spanish. Most participants were female, and about half identified as Latinx and were from Puerto Rico (see Table 1). The greatest attrition occurred between Ws 2 and 3, after the onset of the COVID-19 pandemic.

### 2.2. Measures—Pre-Pandemic Factors

W1 Prior Trauma History. Thirteen items from the Life Events Checklist-5 (LEC-5), a 17-item self-report measure designed to assess exposure to a variety of potentially traumatic events in a respondent’s lifetime, were used [56]; events include a fire or explosion, transportation accident, physical or sexual assault, life-threatening illness or injury, or other very stressful experiences, etc. Participants selected whether they experienced each event by selecting no (0) or yes (1). All items were summed into a total composite score. Among a sample of undergraduate students, the LEC-5 was correlated with PTSD symptoms and demonstrated test-retest reliability [56].

W1 Disaster Exposure. Participants responded to disaster exposure questions based on prior disaster MH research [20,57,58,59]. Items varied slightly based on hurricane and wildfire questionnaire versions, but both asked whether participants had experienced threat, loss, damage, or disruption during the disaster or caused by the disaster (e.g., whether participants were injured or forced to evacuate during the disaster, lost their home, an animal, or a loved one, experienced financial losses because of the disaster, or whether the disaster damaged/destroyed items of sentimental, emotional value, etc.). Response options were no (0) and yes (1). Participants also rated the extent of damage to their permanent and college residences caused by the hurricane on a five-point scale from no damage (0) to total loss or destruction (4). This was converted to a dichotomous scale no damage (0) and any damage (1) based on a prior study in which a descriptive analysis revealed that no damage versus any damage reported at all distinguished between MH outcomes rather than the various levels of damage endorsed [60]. Other prior disaster exposure measures demonstrated strong predictive validity of MH outcomes post-disaster (e.g., internalizing symptoms, anxiety, depression, PTSS) among youth and adults [58,59].

In the current study, hurricane and wildfire exposure experiences were summed separately to create a continuous measure of exposure to each type of event. This was because some questions differed slightly depending on whether the hurricane, wildfire, or mudslide exposure questions were asked. Wildfire and mudslide experiences were combined since these events occurred shortly after each other in Santa Barbara, California, in winter 2017 and 2018. If a participant endorsed experiences relating to both the wildfire and the mudslide (e.g., having had to evacuate during both events), then only one point would be allocated, representing both disasters. Items were summed to create a composite disaster exposure score. Disaster exposure sum scores were transformed into *z*-scores by disaster type (hurricane, wildfire only, or wildfire and mudslide) to increase interpretability of exposure across disasters.

W1 Life Stressors Since Disaster. Participants were asked if they had experienced 11 different life stressors in the time since the natural disaster. Items were adapted from questions used in prior research following wildfires [59] and asked about job changes, moving away, illness or injury to self or a family member, money problems, relationship problems, etc. Response options were no (0) and yes (1). Total scores were computed by the sum of the dichotomized items. Prior research found that the disaster exposure sum was significantly and positively associated with the number of life stressors endorsed since the disaster among a sample of youth (*r* = 0.57, *p* < 0.001 [59]); participants who reported greater levels of disaster exposure also endorsed more life stressors since disaster, demonstrating convergent validity for both constructs as measures of the disasters’ impact.

### 2.3. Measures—Pre- and during Pandemic Mental Health

Waves 1–4 Anxiety. The Generalized Anxiety Disorder-7 (GAD-7), a seven-item self-report questionnaire, was used to screen and measure the severity of symptoms of anxiety [61]. Participants indicated how often they experienced anxiety symptoms within the last two weeks (e.g., “feeling nervous, anxious, or on edge”) using a four-point response scale ranging from not at all (0) to nearly every day (3). A total score was formed by summing responses, with higher scores indicating the presence of a possible anxiety disorder. The GAD-7 is a reliable and valid instrument for accurately assessing generalized anxiety disorder symptoms in university students [62] and demonstrates strong criterion, construct, factorial, and procedural validity [61]. Cronbach’s alpha for this measure in the current sample was high. For the first three Ws, α = 0.90, and for W4, α = 0.89.

Waves 1–4 Depression. The Patient Health Questionnaire-9 (PHQ-9) was used to measure depressive symptoms and consists of a nine-item self-report measure of depressive symptoms based on DSM-IV criteria [63]. Respondents were asked how often they had experienced depressive symptoms within the last two weeks (e.g., “feeling down, depressed, or hopeless”); response options ranged from not at all (0) to nearly every day (3). Total sum scores were calculated. The PHQ-9 has demonstrated good internal reliability and test-retest reliability [64] and has also been shown to assess depression accurately and sustainably among 857 diverse racial/ethnic U.S. university students [65]. Cronbach’s alpha in the current sample for Ws 1–4 was α = 0.89, 0.89, 0.90, and 0.89, respectively, indicating strong internal consistency.

Waves 1–4 Posttraumatic Stress (PTSS). The PTSD Checklist for DSM-5 (PCL-5) is a 20-item self-report measure that assesses DSM-5 symptoms of PTSD and was used in the current study to measure PTSS [66]. It is intended to screen for PTSD, monitor symptom change over time, and make a provisional PTSD diagnosis. The PCL-5 requires that respondents refer to a stressful or traumatic experience and its impact when thinking about problems they may have experienced within the last month. Ws 1 and 2 instructed participants to think about their experiences with natural disasters when answering the questions, while Ws 3 and 4 instructed participants to think about their experiences related to the COVID-19 pandemic. Some sample items include, “In the past month, how much were you bothered by: repeated, disturbing, and unwanted memories of the stressful experience (natural disaster or the COVID-19 pandemic); feeling very upset when something reminded you of the stressful experience; avoiding memories, thoughts, or feelings related to the stressful experience?”, etc. The self-report rating scale ranges from not at all (0) to extremely (4). A total sum score was computed. The PCL-5 has demonstrated strong psychometric properties among trauma-exposed undergraduate students [62], including internal consistency, test-retest reliability across testing occasions, convergent validity, and discriminant validity [67]. Cronbach’s alpha in the current sample for Ws 1–4 was α = 0.93, 0.90, 0.94, and 0.93, respectively, indicating strong internal consistency.

### 2.4. Analytic Procedure

The following preliminary analyses were conducted: descriptive statistics, Pearson’s correlations, independent samples *t*-tests (to examine differences by sex and region based on previous findings [17] and W1-only and extended study participants), and one-way analysis of variance (ANOVA) tests (to examine differences among ethnicity groups and disaster type/region). Power analyses were also conducted using G*Power, Release 3.1.9.7 [68] and the Repeated Measures and Sample Size (RMASS) online power calculator, designed for two-level repeated measure designs and able to account for attrition rate [69,70]. To achieve a power level of 0.9, a sample of 198 participants was required; thus, the current study’s longitudinal sample of 205 young adults was deemed adequate. Next, each MH trajectory was plotted using IBM SPSS Statistics for Macintosh, Version 28.0 [71] to examine its visual shape and inform whether linear, quadratic, and/or cubic trends could be imposed [72].

To test the current study aims, hierarchical linear modeling (HLM) was conducted using HLM8 for Windows, Version 8.0 [73]. Level-one time variables representing MH symptom trajectory patterns across waves were created and coded as follows: linear shape (W1 = 0, W2 = 1, W3 = 2, W4 = 3), quadratic shape (W1= 0, W2 = 1, W3 = 4, W4 = 9), and cubic shape (W1 = 0, W2 = 1, W3 = 8, W4 = 27). Level-two person variables included pre-pandemic factors (e.g., prior trauma history, disaster exposure, life stressors since disaster) and demographics (e.g., sex, region). The following procedures were repeated three separate times for each MH symptom trajectory (anxiety, depression, and PTSS). HLM assumptions were examined and if unmet, adjustments were made. First, a null model was conducted. Second, three models with level-one time variables added one at a time (e.g., linear only, then linear and quadratic, and finally linear, quadratic, and cubic) were conducted. Improvements in model fit were examined. Significant level-one variables were retained and included in subsequent analyses. Third, all level-two person variables were simultaneously entered into the next model; fit was examined, and significant level-two variables were retained. Finally, an additional model with cross-level interaction terms included (e.g., between level-one time and level-two person variables) was conducted if significant changes in MH symptoms were found across waves. Varying degrees of level-two person variables (e.g., low and high levels of W1 disaster exposure) were examined as predictors of MH symptom changes (i.e., trajectory growth rates). Model fit was examined, and significant interaction terms retained. Non-significant terms were eliminated to create a final, parsimonious model. 

Post-hoc analyses examined participants’ MH symptom severity. Anxiety, depression, and PTSS total sum scores were calculated at each wave. Lower scores indicate lower symptom severity, and higher scores indicate higher symptom severity. Anxiety and depressive total sum scores of 10 or above [61,63,74] and PTSS total sum scores of 31–33 or greater [75] are clinically meaningful and indicate possible generalized anxiety disorder, clinical depression, or PTSD, respectively. In these cases, further assessment by an MH professional is recommended.

## 3. Results

### 3.1. Preliminary Analyses

Most pre-pandemic factors and MH outcomes were associated. See Supplementary Materials, Tables S1–S3, for descriptive statistics and Pearson’s correlations. At W1, young adults reported experiencing on average at least two prior traumatic events in their lifetime (*M* = 2.45, *SD* = 2.05) and under three life stressors since disaster (*M* = 2.78, *SD* = 2.54). Across all waves, average anxiety and depression sum scores ranged from 6.66 to 7.27 and from 7.63 to 8.30, respectively, and the average PTSS total scores ranged from 10.14 (at W1) to 20.66 (at W3). Female sex was significantly associated with W3 anxiety and W3 PTSS. Region (Puerto Rico) was significantly associated with W1 life stressors since disaster, W3 anxiety, Ws 1–3 depression, and Ws 1–3 PTSS. Young adults who participated in the current longitudinal study reported greater prior trauma history and life stressors since disaster and more severe MH symptoms than W1-only participants (see Table S4).

### 3.2. Hierarchical Linear Modeling Analyses

**Figure 2:**
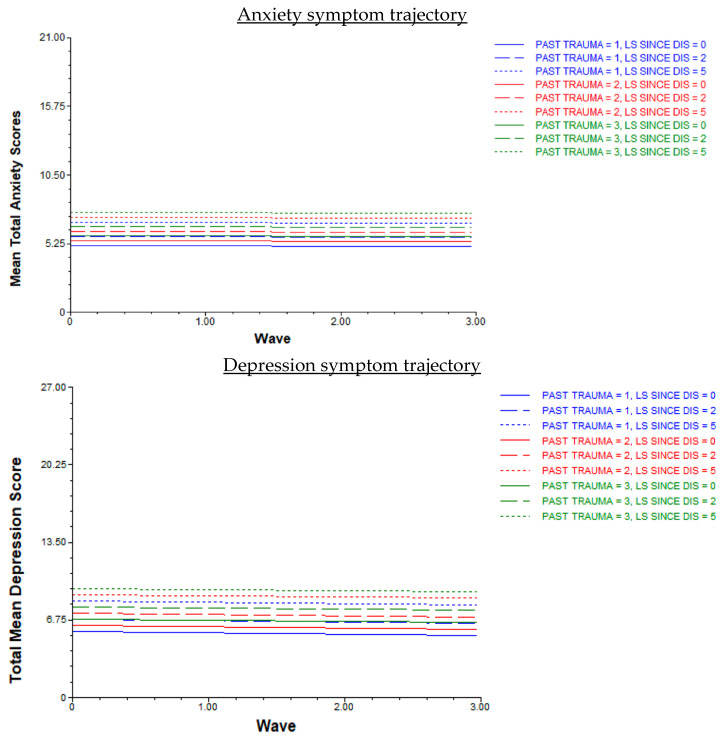
Disaster-exposed young adult anxiety and depression symptom trajectories pre- to during the COVID-19 pandemic (*n* = 205). Note. PAST TRAUMA = prior trauma history; LS SINCE DIS = life stressors since the disaster; Waves (Wave 0 = W1; Wave 1.00 = W2; Wave 2.00 = W3; Wave 3.00 = W4).

Anxiety Trajectory. The null anxiety model intra class correlation (ICC) statistic indicated significant variation among W1 anxiety symptoms (trajectory intercept; *ß* = 6.65, *SE* = 0.35, *t* = 18.98, *p* < 0.002), with 58.9% of the proportion explained by level-two factors. The anxiety trajectory shape was linear and did not significantly differ across waves (*ß* = −0.02, *SE* = 0.14, *t* = −0.13, *p* = 0.897). Prior trauma history (*ß* = 0.39, *SE* = 0.19, *t* = 2.04, *p* < 0.042) and life stressors since disaster (*ß* = 0.36, *SE* =.16, *t* = 2.23, *p* < 0.027) were significantly associated with W1 anxiety (trajectory intercept). In sum, anxiety symptoms did not change and on average, remained stable across all pre- and during-pandemic time points. Because of this, associations between cross-level interaction terms (i.e., between level-one time and level-two person variables) and changes in MH symptoms between waves (i.e., trajectory growth rates) were not examined. Young adults that experienced greater amounts of prior trauma and life stressors since disaster reported more severe W1 anxiety symptoms than those that experienced less prior trauma and fewer life stressors (see Figure 2); W1 anxiety symptom levels were sustained across waves.

Depression Trajectory. The null depression model ICC statistic indicated that 61.3% of the proportion of variance in depression symptoms was accounted for by level-two person characteristics. The null model also revealed that the homogeneity assumption of level-one variance was not met (χ^2^ = 262.41(188), *p* < 0.001). A second model was conducted with sex included as part of the level-one error term, which resulted in a heterogenous covariance model and met the assumption of homogeneity test. This adjusted null depression model was retained in all subsequent analyses. There was significant variation among W1 depression (trajectory intercept; *β* = 8.01, *SE* = 0.40, *t* = 19.96, *p* < 0.001). The depression trajectory shape was linear and did not significantly differ across waves (*β* = −0.09, *SE* = 0.16, *t* = −0.58, *p* = 0.560). Prior trauma history (*β* = 0.55, *SE* = 0.21, *t* = 2.60, *p* = 0.010) and life stressors since disaster (*β* = 0.54, *SE* = 0.19, *t* = 2.87, *p* = 0.005) were significantly associated with W1 depression (trajectory intercept). Since depression symptoms were stable across waves, associations between cross-level interaction terms and changes in symptoms were not examined. Similar to the anxiety model, young adults that experienced greater amounts of prior trauma and life stressors since disaster reported more severe depression symptoms at W1 compared to those that experienced lower amounts (see Figure 2); W1 depression symptoms were sustained across waves.

**Figure 3:**
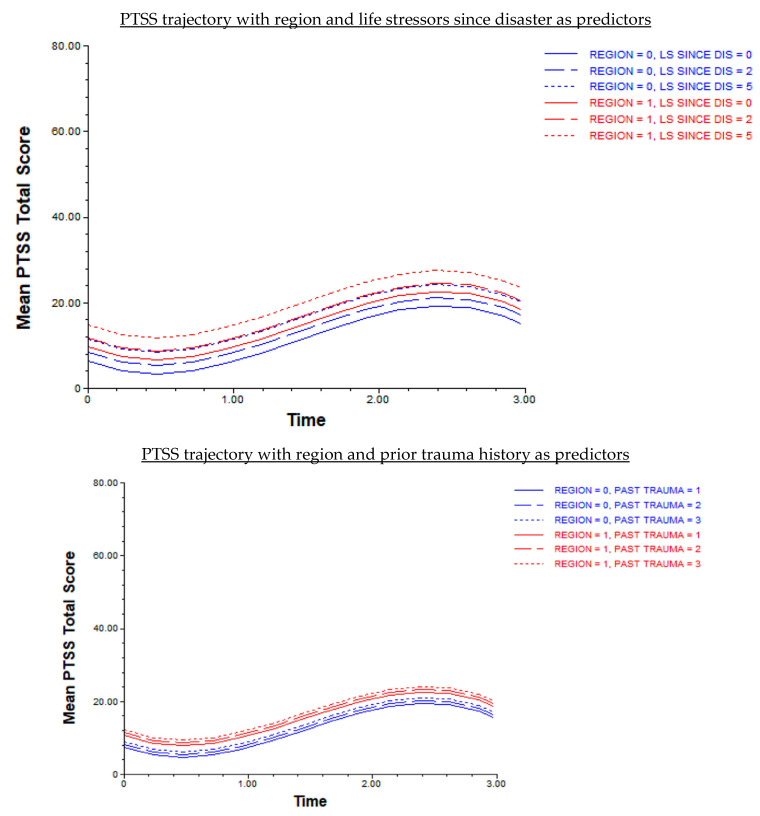
Disaster-exposed young adult post-traumatic stress symptom trajectory pre- to during COVID-19 pandemic (*n* = 205). Note. Region (0 = mainland U.S.; 1 = Puerto Rico); PAST TRAUMA = prior trauma history; LS since dis = life stressors since disaster; Waves (Time 0 = W1; Time 1.00 = W2; Time 2.00 = W3; Time 3.00 = W4).

Post-traumatic Stress Symptoms (PTSS) Trajectory. The null PTSS ICC indicated that 44.8% of the proportion of variance was accounted for by level-two person characteristics. The null model also revealed that the homogeneity assumption of level-one variance was not met. A second model was conducted with sex included as part of the level-one error term, which resulted in a heterogenous covariance model and met the assumption of homogeneity test (see Supplementary Materials, Table S4). This adjusted null PTSS model was used in subsequent analyses. There was significant variation among W1 PTSS (trajectory intercept) and PTSS slopes between waves (trajectory growth rates). The PTSS trajectory shape was cubic (e.g., like the letter *S)* and significantly differed across waves. PTSS decreased between Ws 1–2, sharply increased between Ws 2–3, and gradually decreased between Ws 3–4. The greatest increase in PTSS was observed between Ws 2 and 3 (pre-pandemic and during-pandemic time points). Region (Puerto Rico), prior trauma history, and life stressors since disaster were significantly associated with W1 PTSS (trajectory intercept); pre-pandemic factors and region were not associated with changes in PTSS between waves (trajectory growth rates). Disaster exposure was neither associated with W1 PTSS trajectory intercept, nor growth rates. The random effects for all level-one time variables were significant (see Table S6), which indicated that variation among W1 PTSS and PTSS growth rates was due to other person-level factors that were not accounted for in the current study. These random effects were retained in subsequent models. Interaction terms between level-one and level-two variables were not significant. See Table 2 for the final model results. No associations were found between cross-level interaction terms and PTSS growth rates between waves. Young adults from Puerto Rico and those that experienced greater prior trauma history and life stressors since disaster reported more severe W1 PTSS symptoms compared to young adults in the mainland U.S. and/or those with lower amounts of prior trauma and life stressors post-disaster (see Figure 3). On average, young adults that reported more severe W1 PTSS, also endorsed more severe PTSS at each wave of the trajectory.

### 3.3. Post Hoc Analyses

Across all waves, 23.7% to 30.8% of young adults had total sum anxiety scores at or above the clinical cutoff, indicating a need for further assessment by a MH professional. The highest frequency of individuals at or above the anxiety clinical cutoff score was at W3 (summer 2020, about six months after the pandemic began), while the lowest frequency was at W4 (spring 2021, about one year after the pandemic began). Over a quarter of young adults had total sum depression scores at or above the clinical cutoff with frequencies across waves as follows: W1 (30.7%), W2 (32.3%), W3 (35.9%), and W4 (34.4%). The average total sum depression score at each wave fell between five and ten, suggesting that the sample experienced mild to moderate depressive symptoms throughout the study period. Across Ws 1 and 2, a minority of young adults in the current study met probable criteria for PTSD (4.4% and 5.1%, respectively); however, this increased from W2 to W3 (pre- to during-pandemic). At W3, almost a quarter (22.3%) of young adults met probable criteria for PTSD, which reduced to 17.4% at W4. Average total sum PTSD scores for young adults in the current sample were as follows: W1 (*M* = 10.14, *SD* = 10.38), W2 (*M* = 10.46, *SD* = 10.34), W3 (*M* = 20.66, *SD* = 16.31), and W4 (*M* = 17.91, *SD* = 14.51). Changes in PTSD scores of ten points or greater are considered clinically significant; a ten-point increase was observed between Ws 2 and 3.

### 3.4. Summary

Anxiety and depressive symptom trajectories were linear, and did not differ across waves (from pre- to during-pandemic time points). The PTSS trajectory was *S*-shaped or cubic, meaning that symptoms fluctuated and differed at each wave. PTSS slightly decreased between Ws 1 and 2 (pre-pandemic time points), sharply increased between Ws 2 and 3 (summer 2020, during-pandemic), and began to gradually decrease between Ws 3 and 4 (spring 2021, during-pandemic). Prior trauma history and W1 life stressors since the disaster were the only pre-pandemic factors significantly associated with each MH trajectory intercept (W1 anxiety, W1 depression, and W1 PTSS); region, reported as living in Puerto Rico at W1, was the only demographic characteristic significantly associated with the PTSS trajectory intercept (W1 PTSS). No pre-pandemic or demographic factors were associated with changes in PTSS across waves (trajectory growth rates). 

## 4. Discussion

The current study conducted HLM analyses utilizing prospective and longitudinal data to examine MH symptom trajectories and associations with pre-pandemic factors among disaster-exposed young adults in the mainland U.S. and Puerto Rico. First, results indicated that anxiety and depressive symptom trajectories did not change from pre- to during-pandemic time points, while PTSS fluctuated between each wave. PTSS sharply increased between pre-pandemic to during-pandemic time points. These trajectory patterns suggest that disaster-exposed young adults may experience temporary increases in PTSS, but not anxiety or depression after exposure to a subsequent disaster. Second, young adults with a greater amount of prior trauma history and life stressors since disaster experienced more severe MH symptoms at W1, suggesting that trauma and life stress are risk factors associated with post-disaster MH at proximal time points (W1). Pre-pandemic factors were not associated with later changes in PTSS, suggesting that distal factors may not predict MH symptom changes after exposure to a subsequent disaster. Other proximal factors (e.g., related to the COVID-19 pandemic) may be associated with increases in PTSS during the pandemic, rather than pre-pandemic factors (e.g., those associated with prior natural disaster exposure). Interpretation of results and clinical implications are discussed.

### 4.1. Mental Health Trends Pre- and during Pandemic

The current study findings contribute to the limited research examining long-term MH symptom patterns among disaster-exposed young adults [12]. After disaster exposure, resilience and adaptation are common among most adults, and typical recovery trajectories demonstrate gradual declines in distress and MH symptom severity over time [2,12,75,76]. Our results differ from prior research and suggest that disaster-exposed young adults may experience sustained anxiety and depression and increases in PTSS when exposed to a subsequent disaster. The occurrence of the COVID-19 pandemic (and associated trauma exposure and stressors) may have interrupted expected declines in anxiety and depression post-natural disaster and contributed to increases in PTSS. The gradual decline in PTSS in 2021 suggests that the current sample of disaster-exposed young adults may have begun to adjust to the pandemic.

Several studies examined anxiety and depression symptoms pre- and during-pandemic among young adults; however, because the current sample was recruited based on exposure to natural disasters, it is difficult to make direct comparisons with non-disaster exposed groups. Meta-analyses found substantially higher rates of MH symptoms and disorders among individuals exposed to natural disasters compared with those who were not [1,77]. Among a community sample of young adults (not recruited based on disaster exposure), significant increases in anxiety, depression, and PTSS [49,78,79,80] were observed from pre- to during-pandemic time points (spring 2020); on average, these increases in symptoms began to decline significantly by summer 2020 [81]. We do not know if our sample experienced temporary increases in MH symptoms (e.g., particularly anxiety and depression) in spring 2020 that gradually returned to pre-pandemic levels since the current study’s first wave of pandemic data collection occurred in summer 2020.

While there is limited research on changes in anxiety and depression symptom trajectories after exposure to an initial and subsequent disaster, the PTSS patterns we observed are consistent with other findings. Exposure to a prior disaster is associated with increases in PTSS during the occurrence of a subsequent disaster (e.g., adults exposed to the 11 September 2001 terrorist attack and Hurricane Sandy in 2012; brush fires in 2019–2020 and the COVID-19 pandemic in 2020) [3,4,5,82,83,84,85]. Exposure to multiple disasters may have cumulative and additive effects on MH symptoms [3,4,5] and heighten emotional reactions. Adults exposed to multiple disasters report more severe MH symptoms (e.g., PTSD, depression) than those exposed to a single disaster [3].

Lastly, within the current study, up to 30% of disaster-exposed young adults experienced clinically significant MH symptoms. This is consistent with prior MH disaster studies [2] and indicates a need for psychological support post-disaster. Our sample endorsed having mild anxiety and mild to moderate depression across all waves (pre- to during-pandemic). Roughly 5% of the sample endorsed clinically significant PTSS post-natural disaster, but after the start of the pandemic, this increased to 22.3%. It is important to acknowledge that there are sex differences in MH symptoms (e.g., anxiety, depression); however, because the current study sample was small and mostly female, we had limited ability to address this and control for sex. Examining underlying factors that may explain sex differences, such as the use of rumination as an emotional regulation strategy [86], may be important, as these tendencies are modifiable and can be targeted through interventions.

#### 4.1.1. Prior Trauma History and Life Stressors since Disaster

Only prior trauma history and W1 life stressors since disaster were associated with W1 anxiety, W1 depression, and W1 PTSS trajectory intercepts, and no pre-pandemic factors were associated with changes in PTSS between waves (trajectory growth rates). These results suggest that prior trauma history and life stressors since disaster are associated with PTSS severity at W1, a time point that is most proximal to when the natural disasters occured; data were collected three months to one-and-a-half years post-disaster. Young adults that experienced greater prior life trauma and life stressors since disaster may experience more severe MH symptoms that are sustained across waves compared to those with lower amounts of prior trauma or life stressors since disaster; however, these factors were not associated with changes in PTSS that were observed before and during the COVID-19 pandemic. Proximal factors relating to life circumstances and experiences during the pandemic (e.g., COVID-19-related life stressors), rather than distal pre-pandemic factors, may have been associated with increases in PTSS at W3 but were not examined in the current study. Disaster exposure was not associated with any MH trajectory intercept or PTSS growth rates, and may serve as a proxy for the range of experiences that occur during the time-limited duration of a disaster (e.g., threat to safety), but do not account for life stressors that emerge post-disaster. Existing research suggests that life stressors since disaster may have a greater and long-term impact on socio-emotional health than disaster exposure [58]. Among youth with moderate to low disaster exposure, endorsement of greater life stressors since disaster was associated with lower levels of belief in self and others and lower levels of emotional competence.

Experiences during a disaster can vary by event and region; thus, it is important to consider characteristics of the specific disaster event and relevant cultural and socioecological factors (e.g., systemic racism, pre-existing health disparities, poverty, historical practices) that can affect recovery processes and post-disaster MH. Racial/ethnic minority groups (e.g., Black, Latinx, and indigenous communities) are often most negatively impacted by disasters [2]. Young adults living in Puerto Rico reported more severe W1 PTSS than those in mainland U.S., which was sustained throughout waves. Puerto Rico has been an unincorporated territory of the U.S. since 1898 and has experienced chronic, compounded effects of multiple disasters specific to the island. Some examples include economic difficulties and a debt crisis resulting from austerity policies, sociopolitical tension and turmoil, Dengue fever and Zika virus outbreaks, the destruction caused by Hurricanes Irma and Maria in September 2017, and ongoing earthquakes since December 2019, which have caused displacement in southwest regions of the main island [87]. Historically, Puerto Rico has also received limited government and humanitarian aid during post-disaster recovery periods [87]. The occurrence of many potentially traumatic events in the region and the resulting life stressors may partially explain why PTSS is higher for those in Puerto Rico than the mainland U.S. Additional research is needed to better understand and identify factors associated with post-disaster MH among young adults living in Puerto Rico. For example, cultural factors such as *familismo* buffered against severe pandemic-MH symptoms among Latinx young adults [36,88,89]; however, such protective factors are less commonly examined post-disaster [90].

#### 4.1.2. Implications for Practice

The current study’s findings provide information about MH trends among young adults exposed to multiple disasters and identify those at greater risk of experiencing more severe MH symptoms. MH and medical professionals that work with young adults should assess prior exposure to disasters when assessing for overall trauma exposure. Young adults’ MH symptom levels should also be routinely monitored post-disaster over extended periods of time. Sustained mild to moderate MH symptoms or clinically significant increases in symptom severity (e.g., PTSS during a subsequent disaster) may warrant psychological support. MH professionals should keep in mind that young adults with a greater amount of prior trauma exposure or life stressors after an initial disaster and/or that identify as part of an ethnic/racial or other minority group may experience more severe PTSS symptoms during a subsequent disaster. Efforts should be made to connect young adults that are most vulnerable or at-risk to accessible, low-cost MH services. It may also be important to provide psychoeducation about possible emotional experiences post-disaster, which may normalize a variety of responses. Young adults can monitor/check-in on their own personal reactions and those of their peers.

Important prevention/intervention efforts include psychological first aid, which provides psychosocial support in the immediate aftermath of a traumatic event and cognitive-behavioral therapeutic modalities, both of which were associated with reduced MH symptom severity and distress after disasters such as the 11 September 2001, terrorist attack and Hurricane Katrina [91,92,93,94]. In addition to focusing on disaster exposure experiences themselves, MH providers working with disaster-exposed young adults should consider exploring personal identity and intersectionality, experiences of social injustice [87,95], and young adult psychosocial development [15], which can impact MH. While COVID-19 was no longer declared a public health emergency in the U.S. on 11 May 2023, the pandemic may have long-term impacts on many life domains (e.g., health, financial, developmental) and has further perpetuated disparities among communities of color. It is important to consider how the pandemic affected young adults during acute phases and may continue to do so in present day.

Finally, fostering and/or bolstering social support and social capital among young adults and their broader communities (e.g., neighborhoods, universities) can be protective in post-disaster contexts [36,95,96]. Universities can play an important role in supporting disaster-exposed young adult university students. Some examples may include providing or connecting students to needed instrumental support (e.g., housing, food, financial aid [96]), improving access to psychological support, facilitating campus events (e.g., activities, memorials, vigils, psychological support groups) to promote social connectedness/unity and encourage meaning-making, allowing academic accommodations as necessary, and facilitating academic and career-related mentorship and training.

#### 4.1.3. Strengths and Limitations

The current study has notable strengths. First, this multi-site, multi-disaster longitudinal study collected data focused solely on the experiences of young adults exposed to natural disasters in 2017 and 2018, and the COVID-19 pandemic in 2020. Second, prospective data was utilized to uniquely examine longitudinal associations between pre-pandemic factors and during-pandemic MH symptoms. The current study also provided an opportunity to examine long-term MH symptom patterns after initial and subsequent exposure to collectively experienced traumatic events.

There are also many limitations to this study. High levels of attrition occurred between each wave which may reduce the strength of the results. Over half of W1 study participants (*n* = 466) provided consent to be contacted and invited to participate in the extended study; however, only 36% of those participants (*n* = 169) completed the W2 survey, indicating difficulty retaining participants. Significant differences between W1-only and extended study participants were also found, suggesting that the current study results may be biased. Compared to W1-only participants, extended study participants reported significantly greater prior trauma history, W1 life stressors since disaster, and W1 MH symptom severity. Extended study participants may have experienced greater impacts related to prior trauma history, life stressors since disaster, etc., which may have increased motivation to participate in Ws 2–4. 

In addition, the current study sample was mainly female, greatly limiting generalizability. Sexual orientation was also not included as a primary demographic factor. It is important that future disaster MH research include young adults of all sexual and gender identities, including individuals that identify as being a member of a sexual and/or gender minority group. The current study sample also only included young adults that were attending a four-year university at W1, omitting non-university attending young adults. Efforts to recruit and retain diverse participants in longitudinal disaster MH studies is critical to include a variety of experiences and viewpoints, and increase understanding of the long-term impacts of disaster. Lastly, incorporating a qualitative component or mixed methods approach to the current research would have provided greater depth to disaster-exposed young adults’ experiences. Asking specific questions about contextual, cultural, or sociopolitical factors and their associations with MH post-disaster could also aid with accurate interpretation and understanding of possible regional differences (e.g., Puerto Rico and/or the mainland U.S.).

## 5. Conclusions

Identifying long-term MH patterns and prospective factors associated with symptom severity can help to predict subsequent MH outcomes after future collectively experienced traumatic events. Findings suggest that young adults exposed to a natural disaster may experience sustained anxiety and depression symptoms and increases in PTSS after exposure to an additional, later-occurring collective trauma (e.g., the COVID-19 pandemic). Young adults with greater prior trauma history and life stressors since disaster may experience more severe MH symptoms post-disaster. MH professionals, university staff, and community members in regions affected by multiple disasters can use these findings to identify and support at-risk young adult university students that may benefit from psychological support or other interventions aimed at promoting adaptation and recovery post-disaster.

## Figures and Tables

**Table 1:** Longitudinal participant demographics (*n* = 205).

	*n*	%
Sex		
Female	177	86.3
Male	28	13.7
Ethnicity		
Asian	24	11.7
Black	9	4.4
Latinx	102	49.8
White	49	23.9
Mixed/Other	21	10.3
Missing	1	0.5
Region by Disaster		
Mainland U.S.		
Hurricane	50	24.4
Wildfire	58	28.3
Puerto Rico		
Hurricane	97	47.3
Region		
Mainland U.S.	108	25.7
California	58	28.8
Florida	42	20.5
Texas	8	3.9
Puerto Rico	97	47.3
Wave Participation		
Wave 1	205	-
Wave 2	162	79
Wave 3	117	57.1
Wave 4	97	47.3
Attrition Rate		
Waves 1–2	43	21
Waves 2–3	45	27.8
Waves 3–4	20	17.1
W1 University Class		
Freshman	44	21.5
Sophomore	44	21.5
Junior	28	13.7
Senior	48	23.4
Graduate Student	39	19
Missing	2	1
	*M*	*SD*
W1 Age	21.38	3.27

Note. This table includes participants who completed W1 and one or more later waves.

**Table 2:** Parameter estimates of level-one and level-two predictors of PTSS trajectory (*n* = 205).

Model 7 Predictors of PTSS Trajectory					
Fixed Effects	Coeff.	SE	t	df	*p*-Value
Initial Status in Model					
M of Initial Status	4.13	1.03	4.00	201	<0.001
Region	3.06	1.40	2.18	201	0.030
Trauma History	0.76	0.37	2.04	201	0.042
Life Stressors Since Disaster	0.93	0.31	3.01	201	0.003
Change Rate in Model				199	
M of Linear Growth Rate	−12.65	2.34	−5.40	204	<0.001
M of Quadratic Growth Rate	16.42	2.61	6.29	204	<0.001
M of Cubic Growth Rate	−3.78	0.63	−6.00	204	<0.001
Random Effects	Variance			*df*	*p*-value
Intercept	64.00			44	<0.001
Linear Growth	300.57			49	<0.001
Quadratic Growth	452.93			49	<0.001
Cubic Growth	27.68			49	<0.001
Deviance Statistic (Parameters)	4162.76 (19)				
Comparison	χ^2^(*df*)				*p*-value
Model 6	20.91(13)				0.070
Model 5	40.61(3)				<0.001

Note. Coeff. = coefficient. Region (0 = mainland U.S.; 1 = Puerto Rico); Sex (0 = Male; 1 = Female).

## Data Availability

The data presented in this study are available on request from the corresponding author. The data are not publicly available due to privacy and ethical considerations and needing to store/share data in accordance with consent provided by participants.

## Supplementary Materials

The following supporting information can be downloaded at https://www.mdpi.com/article/10.3390/bs14090787/s1, Table S1. Descriptive statistics of level-two person characteristics and anxiety (*n* = 205); Table S2. Descriptive statistics of level-two person characteristics and depression (*n* = 205); Table S3. Descriptive statistics of level-two person characteristics and PTSS (*n* = 205); Table S4. Results of the PTSS null and heterogenous model accounting for level-one homogeneity by sex (*n* = 205); Table S5. Parameter estimates of level-one time predictors of PTSS trajectory pre- to during pandemic (*n* = 205); Table S6. Parameter estimates of level-two person characteristics and level-one time predictors of PTSS trajectory (*n* = 205).
