# Supplementary material for: A Prospective Examination of Mental Health Trajectories of Disaster-Exposed Young Adults in the COVID-19 Pandemic
Source: Behav Sci (Basel). 2024 Sep 7;14(9):787. doi: 10.3390/bs14090787 (PMC11428824; doi:10.3390/bs14090787)
Supplement: Supplementary file 1 [file behavsci-14-00787-s001.zip › behavsci-3037079-supplementary.pdf]

Supplementary Materials

Table S1.

Descriptive Statistics of Level-Two Person Characteristics and Anxiety (n = 205)

Variable	1	2	3	4	5	6	7	8	9
1. Sex [a]	-								
2. Region [b]	.18*	-							
3. Prior Trauma History	-.01	.06	-						
4. W1 Dis Exposure	-.12	-.13	.22**	-					
5. W1 LS Since Dis	.10	.50**	.27**	.25**	-				
6. W1 Anxiety	.07	.16*	.20**	-.02	.21**	-			
7. W2 Anxiety	.05	.12	.13	.01	.21**	.59**	-		
8. W3 Anxiety	.24*	.23*	.19*	-.05	.24*	.63**	.67**	-	
9. W4 Anxiety	.18	-.01	.17	-.06	.27**	.53**	.50**	.47**	-
M	-	-	2.45	.12	2.78	6.66	6.69	7.27	6.37
SD	-	-	2.05	1.02	2.54	5.20	5.09	5.53	4.79

Note. DIS = disaster; LS = life stressors. Total scores were used for Waves 1-4 anxiety. [a]0 = male and 1 = female. [b]0 = mainland U.S. and 1 = Puerto Rico. * $p < .05$, ** $p < .01$.

Table S2.

Descriptive Statistics of Level-Two Person Characteristics and Depression (n = 205)

Variable	1	2	3	4	5	6	7	8	9
1. Sex [a]	-								
2. Region [b]	.18*	-							
3. Trauma History	-.01	.06	-						
4. W1 Dis Exposure	-.12	-.13	.22**	-					
5. W1 LS Since Dis	.10	.50**	.27**	.25**	-				
6. W1 Depression	-.01	.27**	.18**	-.01	.32**	-			
7. W2 Depression	.02	.16*	.27**	-.05	.27**	.63**	-		
8. W3 Depression	.10	.24**	.23*	-.05	.28**	.58**	.76**	-	
9. W4 Depression	.05	.15	.26*	-.02	.32**	.53**	.57**	.60**	-
M	-	-	2.45	.12	2.78	8.08	7.94	8.30	7.63
SD	-	-	2.05	1.02	2.54	5.90	5.93	6.31	5.50

Note. DIS = disaster; LS = life stressors. Total scores were used for Waves 1-4 depression. [a]0 = male and 1 = female. [b]0 = mainland U.S. and 1 = Puerto Rico. * $p < .05$, ** $p < .01$.

Table S3.

Descriptive Statistics of Level-Two Person Characteristics and PTSS (n = 205)

Variable	1	2	3	4	5	6	7	8	9
1. Sex [a]	-								
2. Region [b]	.18**	-							
3. Trauma History	-.01	.06	-						
4. W1 Dis Exposure	-.12	-.13	.22**	-					
5. W1 LS Since Dis	.10	.50**	.27**	.25**	-				
6. W1 PTSS	.17*	.31**	.22**	.10	.33**	-			
7. W2 PTSS	.14	.23**	.19*	.14	.35**	.69**	-		

8. W3 PTSS	.20*	.08	.13	.14	.24**	.54**	.57**	-	
9. W4 PTSS	.07	.15	.20	.06	.43**	.47**	.62**	.55**	-
M	-	-	2.45	.12	2.78	10.14	10.46	20.66	17.91
SD	-	-	2.05	1.02	2.54	10.38	10.31	16.31	14.15

Note. PTSS = post-traumatic stress symptoms; DIS = disaster; LS = life stressors. Total scores were used for Waves 1-4 PTSS. [a]0 = male and 1 = female. [b]0 = mainland U.S. and 1 = Puerto Rico. * p < .05, ** p < .01.

Table S4.

Results of the PTSS Null and Heterogenous Model Accounting for Level-One Homogeneity by Sex (n = 205)

Fixed Effects (*M*)	Model 1- Null				Model 2- Heterogeneous			
	Coeff.	*SE*	*t*	*df*	*Coeff.*	*SE*	*t*	*df*
Initial Status	13.14***	.74	17.74	204	13.04***	.73	17.75	204
Level 1-Variance					*Coeff.*	*SE*	*Z*-ratio	df
Initial Status					3.96***	.21	19.25	-
Sex [a]					.65**	.22	2.96	-
Random Effects	Variance			*df*	Variance			*df*
Intercept	76.39***			204	74.52***			204
Level 1- Error	94.25			-	-			-
Goodness of Fit								
Deviance Statistic (Parameters)	4405.41 (2)				4396.80 (4)*			
Homogeneity Test, χ^2(*df*)	393.38 (189)***				86.73 (196)			

Note. Model 2 accounts for sex differences present within the level-1 variance and meets the assumption of homogeneity. *Coeff.* = coefficient. [a]0 = male and 1 = female. ** p* < .05*, ** p* < .01*, *** p* < .001.

Table S5.

Parameter Estimates of Level-One Time Predictors of PTSS Trajectory Pre and During-Pandemic (n = 205)

Fixed Effects (*M*)	Model 3: Linear Time			Model 4: Quad. Time			Model 5: Cubic Time		
	Coeff.	*SE*	*t*	*Coeff.*	*SE*	*t*	*Coeff.*	*SE*	*t*
Initial Status, β_{00}	9.25***	.70	13.25	9.31***	.72	12.93	10.04***	.72	13.87
Linear Growth Rate, β_{10}				3.12***	.94	3.32	-12.45***	2.36	-5.28
Quad Growth Rate, β_{20}				.11	.33	.32	16.20***	2.62	6.17
Cubic Growth Rate, β_{30}							-3.72***	.63	-5.88
Random Effects	Variance		*df*	Variance		*df*	Variance		*df*
Initial Status, r_0	47.47***		204	47.39***		110	80.55***		49
Linear Growth Rate, r_1	5.34			4.37		110	315.06**		49
Quad Growth Rate, r_2				.11		110	465.63***		49
Cubic Growth Rate, r_3							28.25***		49
Goodness of Fit									
Deviance Statistic (Parameters)	4287.94 (7)***			4287.82 (11)			4203.37 (16)***		
Comparison	Model 2			Model 3			Model 4		
$\chi^2(df)$	108.87(3)***			11.65(49)			84.46(5)***		

Note. *Coeff.* = model coefficient. Quad = quadratic. *$p < .05$; **$p < .01$; ***$p < .001$.

Table S6.
Parameter Estimates of Level-Two Person and Level-One Time Predictors of PTSS Trajectory (n = 205)

	Model 6- All Predictors				
Fixed Effects	*Coeff.*	*SE*	*t*	*df*	*p*-value
M of Initial Status	4.13	1.12	3.70	200	<.001
Region	4.62	1.69	2.74	200	.007
Trauma History	.71	.40	1.78	200	.078
Disaster Exposure	.38	.75	.50	200	.618
Life Stressors Since Disaster	.71	.37	1.89	200	.061
Change Rate in Model					
M of Linear Growth Rate	-14.07	3.84	-3.67	200	<.001
Region	1.91	5.38	.36	200	.723
Trauma History	.44	1.13	.39	200	.695
Disaster Exposure	1.85	2.50	.74	200	.460
W1 Life Stressors Since Disaster	-.28	1.10	-.26	200	.797
M of Quadratic Growth Rate	19.46	4.28	4.55	200	<.001
Region	-4.89	5.95	-.82	200	.413
Trauma History	-.63	1.19	-.53	200	.595
Disaster Exposure	-1.18	2.68	-.44	200	.660
W1 Life Stressors Since Disaster	.34	1.19	.28	200	.778
M of Cubic Growth Rate	-4.72	1.04	-4.54	200	<.001
Region	1.14	1.44	.79	200	.431
Trauma History	.17	.28	.61	200	.542
Disaster Exposure	.09	.65	.14	200	.889
W1 Life Stressors Since Disaster	-.01	.29	-.03	200	.977
Random Effects		Variance		*df*	*p*-value
Intercept		63.61		45	<.001
Linear Growth		306.25		45	.003
Quadratic Growth		462.10		45	<.001
Cubic Growth		28.28		45	<.001
Goodness of Fit					
Deviance Statistic (Parameters)			4141.84 (32)		
Comparison to Model 5, $\chi^2(df)$			61.53(16), p <.001		

Note. Coeff. = coefficient. Region (1 = Puerto Rico, 0 = mainland U.S).
